# Nearly Complete Genome Sequence of Curionopolis Virus, a *Culicoides*-Related Rhabdovirus Isolated in the Brazilian Amazon Region

**DOI:** 10.1128/genomeA.01158-14

**Published:** 2014-11-13

**Authors:** Daniele Barbosa de Almeida Medeiros, José Antonio P. Diniz Júnior, Jedson F. Cardoso, Sandro Patroca Silva, Daisy Elaine Andrade da Silva, Layanna F. de Oliveira, Janaina M. é Vasconcelos, Jannifer O. Chiang, Amarilis Aragão Dias, Márcio R. T. Nunes, João Lídio da S. G. Vianez Júnior, Pedro F. C. Vasconcelos

**Affiliations:** a Department of Arbovirology and Hemorrhagic Fevers, Evandro Chagas Institute, Ministry of Health, Ananindeua, Brazil; b Department of Electron Microscopy, Evandro Chagas Institute, Ministry of Health, Belém, Brazil; c Center for Technological Innovation, Evandro Chagas Institute, Ministry of Health, Ananindeua, Brazil

## Abstract

We report here the first nearly complete genome sequence related to curionopolis virus (CURV), that of strain AR440009, isolated from a pool of *Culicoides* sp. midges in Serra Norte, Pará State, northern Brazil. All genes showed similarities to those belonging to members of the family *Rhabdoviridae*.

## GENOME ANNOUNCEMENT

Curionopolis virus (CURV) BE AR440009 was isolated from a pool of *Culicoides* sp. midges captured in Serra Norte, municipality of Parauapebas (50°15’W 6°8’N), Pará State, Brazil, on 12 March 1985 (1). In previous studies, it was shown that this virus belongs to the family *Rhabdoviridae* (2) and has caused neurologic injures in newborn albino Swiss mice (3).

The family *Rhabdoviridae* belongs to the order *Mononegavirales* and is one of the most ecologically diverse families of RNA viruses, infecting a wide range of hosts, such as mammals, marsupials, birds, reptiles, fish, insects, and plants (4) To date, 46 viral species have been recognized and classified in six genera (*Vesiculovirus, Lyssavirus, Ephemerovirus, Novirhabdovirus, Cytorhabdovirus*, and *Nucleorhabdovirus*), five species are unclassified in genus, and 150 ungrouped viruses have not been formally classified (5). In addition, two new genera, *Sigmavirus* (6) and *Tibrovirus* (7), have been proposed for formal classification. The rhabdoviruses are bullet-shaped enveloped viruses with a mean diameter of 45 to 100 nm and length of 100 to 430 nm. Although all rhabdoviruses have single-stranded negative-sense RNA (ssRNA-) and share five canonical structural protein genes (N, P, M, G, and L), several novel and diverse accessory genes have been described whose roles are associated with pathogenesis and apoptosis in animal and cell-to-cell movement in plants (8).

In this study, the CURV particles were precipitated using polyethylene glycol (PEG) centrifugation, as previously described (8), and the supernatant was treated with DNase and RNase (Ambion) for host-contaminant removal. The treated samples were then used for RNA extraction and full-length genome sequencing using a combination of sequencing platforms, GS FLX 454 (Roche Life Sciences) and Ion Torrent PGM (Life Technologies). Regardless of the sequencing platform used, the method used to obtain the genome basically was performed according to the following steps: RNA fragmentation, library preparation (cDNA), emulsion PCR, and sequencing, as previously described (9, 10). The sequencing steps were carried out at the Genomic Core of the Center for Technological Innovation, Evandro Chagas Institute, Brazilian Ministry of Health, Ananindeua, Brazil.

The genome was obtained by employing a *de novo* hybrid assembly strategy using both Ion Torrent and GS FLX 454 reads simultaneously with the software Mira 4.0. Visual inspection was performed with the software Geneious version 6.1.4. The total genome recovered was 13,169 nucleotides (nt) in length, with a mean coverage of 180-fold. The five main genes were recognized, as well as two putative accessory genes (a1 and a2) between the G and L genes, which resulted in the structure 3′-N–P-M-G-a1-a2-L-5'. This is the first report of the complete genome sequence for curionopolis virus, an ungrouped Brazilian *Rhabdoviridae* virus.

### Nucleotide sequence accession number.

The complete genome sequence has been deposited in GenBank under the accession no. KJ701190.
